# Analysis of the equity of emergency medical services: a cross-sectional survey in Chongqing city

**DOI:** 10.1186/s12939-015-0282-8

**Published:** 2015-12-21

**Authors:** Yalan Liu, Yi Jiang, Shenglan Tang, Jingfu Qiu, Xiaoni Zhong, Yang Wang

**Affiliations:** School of Public Health and Management, Chongqing Medical University, the Research Center for Medicine and Social Development, the Innovation Center for Social Risk Governance in Health, Chongqing, China; Duke Global Health Institute, Duke University, Durham, NC USA

## Abstract

**Background:**

Due to reform of the economic system and the even distribution of available wealth, emergency medical services (EMS) experienced greater risks in equity. This study aimed to assess the equity of EMS needs, utilisation, and distribution of related resources, and to provide evidence for policy-makers to improve such services in Chongqing city, China.

**Methods:**

Five emergency needs variables (mortality rate of maternal, neonatal, cerebrovascular, cardiovascular, injury and poisoning) from the death surveillance, and two utilisation variables (emergency room visits and rate of utilisation) were collected from Chongqing Health Statistical Year Book 2008 to 2012. We used a concentration index (CI) to assess equality in the distribution of needs and utilisation among three areas with different per-head gross domestic product (GDP). In each area, we randomly chose two districts as sample areas and selected all the medical institutions with emergency services as subjects. We used the Gini coefficient (G) to measure equity in population and geographic distribution of facilities and human resources related EMS.

**Results:**

Maternal-caused (CI: range −0.213 to −0.096) and neonatal-caused (CI: range −0.161 to −0.046)deaths declined in 2008–12, which focusing mainly on the less developed area. The maternal deaths were less equitably distributed than neonatal, and the gaps between areas gradually become more noticeable. For cerebrovascular (CI: range 0.106 to 0.455), cardiovascular (CI: range 0.101 to 0.329), injury and poisoning (CI: range 0.001 to 0.301) deaths, we documented a steady improvement of mortality; the overall equity of these mortalities was lower than those of maternal and neonatal mortalities, but distinct decreases were seen over time. The patients in developed area were more likely to use EMS (CI: range 0.296 to 0.423) than those in less developed area, and the CI increased over the 5-year period, suggesting that gaps in equity were increasing. The population distribution of facilities, physicians and nurses (G: range 0.2 to 0.3) was relatively equitable; the geographic distribution (G: range 0.4 to 0.5) showed a big gap between areas.

**Conclusions:**

In Chongqing city, equity of needs, utilization, and resources allocation of EMS is low, and the provision of such services has not met the needs of patients. To narrow the gap of equity, improvement in the capability of EMS to decrease cerebrovascular, cardiovascular, injury and poisoning cases, should be regarded as a top priority. In poor areas, allocation of facilities and human resources needs to be improved, and the economy should also be enhanced.

## Background

In recent decades, China has achieved unprecedented economic growth, and the great health achievements are mainly due to economic system reform. However, the available wealth has not been distributed evenly in different regions, leading to a widening gap of health outcomes, resources allocations and utilisation of health services between poor and rich areas [1–7], particularly for the emergency medical services (EMS).

With the aging population increasing and disease spectrum changing, as well as the rapid development of the industry and transportation, the number of casualties was increasing year by year. The main needs of emergency services are from the patients caused by cardiovascular, disasters and accidents. By 2010, the number of patients with diagnosed hypertension in China exceeded 200 million [8]. It was estimated that cardiovascular diseases accounted for 41 % of all deaths in China [9]. According to Chongqing Health Statistical Year Book 2012, cerebrovascular and cardiovascular has sorted the top three causes of resident death and accounted for 17.51 and 17.49 % respectively, while injury and poisoning that mainly motor vehicle traffic accidents, falls and suicide was the fourth causes of death, holding 8.38 % of all deaths, and also part of that are mainly due to limited timely, effective emergency services for losing the chance of survival, especially in the poor areas.

The elderly are an ever-increasing population in overcrowded emergency departments, and their complex medical and social needs require more time and medical resources [10]. It is widely agreed that the current disease-oriented, episodic model does not adequately address the complex needs of older patients [11, 12]. The Montreal Jewish General Hospital in Canada has tested an emergency department geriatric consultation team staffed by a full-time nurse clinician, part-time physical and occupational therapists [13, 14]. A hospital in Turin has reserved two to four Emergency Medicine beds managed by geriatric nurses and geriatricians for older patients [15].

Research showed that pre-hospital mortality varies greatly depending on medical conditions: it may account for over 80 % of all trauma-related fatalities for adults and 60 % for small children, but only a third of fatalities are related to myocardial infarction. Globally, it has been noted, “For every woman who dies, at least 30 others are injured and disabled [16].” As we know, different districts have different mortalities due to different pre-hospital emergency conditions. The area with better EMS condition may have a lower mortality rate and no longer needs more healthcare professionals. Thus, the relationship between mortality rate and EMS needs is complicated in different districts and it should be discussed.

Studies revealed the causes that patients did not seek or use health services in the case of emergency for reasons including lack of financial support, knowledge, resources or technology [17–19], and that patients’ use of services tended to decline with distance or transport costs [20–22]. The emergency utilisation and resources allocation are even more serious in poor regions, and there is significant difference between urban and rural areas in Chongqing. However, the aim of EMS is to provide acute intervention and timely health care to all patients with emergent or urgent problems [23]. We did not find many studies about how EMS equity is evolving over time from various emergency needs of patients and provision of emergency departments.

This study aims to comprehensively analyses trends of equity in EMS needs and utilisation in different economic areas from 2008 to 2012, and to discuss the correlation of EMS needs and provision, then put forward feasible proposals to improve the equity of EMS in Chongqing city.

## Methods

### Data sources

In the study, we used mortality reflecting the EMS needs and the data was obtained from the death surveillance in all 38 districts, which used unified cards and was conducted by Chongqing Center for Disease Control and Prevention.

For the allocation of human resources, we used stratified random cluster methods. All districts were divided into three socio-economic groups by sorting based on the average of per-head GDP in Chongqing city. The groups were, in ascending order, the less developed area, the relatively developed area and the developed area, and the geographical area for each area were 44,981, 23,294 and 14,089 KM2 (square). In each group, we randomly chose two districts as sample areas, and then all the medical institutions with emergency capability, both in and out of emergency networks, were selected as subjects. There were only 2, 3 (less developed area of most rural districts), 2, 3 (relatively developed) and 6, 9 (developed area of most urban districts) institutions of emergency ability in the six sample areas, respectively. The total of 25 institutions was surveyed, and all the emergency department directors and nurses were interviewed with a self-designed questionnaire. The questionnaire included information about the number of full-time and part-time emergency physicians and nurses, both pre-hospital and in-hospital. It was filled by the emergency department director and nurse together.

The information about the emergency medical institutions was from the website of Chongqing Municipal Health and Family Planning. The EMS room visits, per capita GDP, geographic area and population of the 38 districts were collected from Chongqing Health Statistical Year Book from 2008 to 2013.

### Indicators

The EMS in different characteristic area should have different needs of healthcare services due to different population distribution. For a district with more elderly population, it may need more healthcare professionals to take care cerebrovascular/ cardiovascular disease patients while a district with more workers population, they may need more healthcare professional to take care trauma/injury emergency patients. Different needs in different districts. And for different needs of EMS, as well as different medical conditions, different districts should have different EMS utilisation and resources distribution.

Ten groups of indicators were used as measures of EMS needs, utilisation and resources distribution. According to the characteristics of EMS and the availability of health indicators, we chose mortality rates of maternal, neonatal, cerebrovascular, cardiovascular, injury and poisoning cases as different needs indicators in EMS. The maternal was targeted non-accidental death from conception to postpartum 42 days and living in Chongqing city for more than 1 year. The neonatal was defined as children dying within 28 days after birth. The EMS utilisation indicators were emergency room visits and rates of utilisation, which were mainly from the number of uses. The related resources were facilities and human resources. Facilities targeted the medical institutions with emergency capability both in and out of networks. Human resources targeted emergency physicians and nurses, both full-time and part-time staffs of pre-hospital and in-hospital, as the related resources cannot be separated in most districts by Emergency Medical Service System (EMSS).

### Equity measures

Concentration index (CI) and Gini coefficient (G) were employed to describe regional discrepancies and assess the degree of income-related equity for all indicators [24].

Equity in healthcare has been conceptualised and defined in several ways. Two main forms of health equity are identified: vertical equity (people with greater health needs should receive more healthcare than those with lesser needs, which is an ethical judgment and the measurement remains underdeveloped), and horizontal equity (people with the same healthcare needs ought to have the same chance to access healthcare, irrespective of their socioeconomic status, and technical skills to measure it have been advanced) [3]. Due to the limitation of variables, our study did not use standardisation and manually calculated CI to measure horizontal equity of EMS needs and utilisation through the following formula [25]:

$$ CI={\displaystyle \sum_{t=1}^T\left({P}_t{L}_{t+1}-{P}_{t+1}{L}_t\right)} $$

Where *P* is the cumulative percentage of live births or demographic ranked by economic status, *L* is the cumulative percentage of death or emergency room visits, and t is the number of groups. It lies in the range of −1 to +1, with a negative (positive) value indicating the EMS needs related death rate and utilisation are concentrated among the poor (rich) areas. A CI of zero indicates that there is equity among areas [26–28]. The greater the absolute value of CI, the greater the degree of inequality, which means the worse off for the disadvantaged groups of people.

G was used to measure equity in population and geographic distribution of related resources. It calculated as the ratio of the area between the Lorenz curve and the diagonal line, to the whole area below the 45° line [29]. In this study we used the formula provided by M. Brown and manually calculated it.

$$ {S}_1=\frac{1}{2}{\displaystyle \sum_{i=0}^n\left({Y}_i+{Y}_{i+1}\right)}{X}_{i+1} $$

$$ G=2\times \left(0.5-{S}_1\right) $$

Where S_1_ is the area below the Lorenz curve, Y_i_ is the cumulative percentage of resources and Y_0_ = 0, X_i+ 1_ is the proportion of population or geographic area among each group [30, 31]. It ranges from 0 to +1 and a value of 0 indicates perfect equity in distribution of resources. 0.4 is alert status, less than 0.2 indicates a high equity, 0.2 to 0.3 means a relative equity, 0.3 to 0.4 is reasonable, 0.4 to 0.5 stands for a big gap between groups and greater than 0.5 indicates a highly inequity.

### Ethics statement

The ethics clearance was approved by the Ethics Committee of Chongqing Medical University. All investigators in this survey have obtained written informed consent including the objectives and the procedure of the study. The authors declare no conflict of interest.

## Results

### Equity in EMS needs

Table 1 Summaries EMS needs-related mortality rate for each group from 2008 to 2012. Tables 2 and 3 summaries equity measure for all needs indicators in terms of CI.

**Table 1:** EMS needs indicators among different socio-economic groups, Chongqing, 2008 to 2012

Indicator^b^	Year	Less developed area			Relatively developed area			Developed area		
		Mortality	Deaths		Mortality	Deaths		Mortality	Deaths	
		rate^a^	n	%	rate	n	%	rate	n	%
Maternal	2008	43.74	51	49.04	33.89	34	32.69	24.20	19	18.27
	2009	39.73	49	54.44	24.09	23	25.56	22.54	18	20.00
	2010	37.68	37	42.05	20.45	36	40.90	19.14	15	17.05
	2011	26.96	30	46.88	18.69	18	28.12	18.07	16	25.00
	2012	21.67	25	53.19	14.84	15	31.92	7.27	7	14.89
Neonatal	2008	6.47	755	49.57	4.06	407	26.73	4.60	361	23.70
	2009	5.30	654	44.49	5.02	479	32.59	4.22	337	22.92
	2010	5.81	767	50.10	3.69	442	28.87	4.11	322	21.03
	2011	5.02	559	46.39	3.78	364	30.21	3.18	282	23.40
	2012	4.20	484	44.61	3.33	337	31.06	2.74	264	24.33
Cerebrovascular	2008	3.55	416	17.19	7.98	858	35.45	13.67	1146	47.36
	2009	3.04	359	7.61	9.12	984	20.85	33.19	3376	71.54
	2010	41.74	4952	32.76	31.78	3452	22.84	65.12	6713	44.40
	2011	50.87	6069	23.91	81.47	8009	31.55	103.38	11304	44.54
	2012	79.04	9463	28.56	96.61	10575	31.91	128.44	13098	39.53
Cardiovascular	2008	6.35	743	14.59	18.74	2015	39.56	27.86	2336	45.85
	2009	6.96	821	11.70	20.79	2243	31.96	38.87	3954	56.34
	2010	38.40	4556	26.14	38.94	4229	24.26	83.86	8645	49.60
	2011	54.51	6503	24.13	80.81	8830	32.76	111.30	11620	43.11
	2012	88.49	10594	27.01	130.21	14254	36.34	141.01	14380	36.65
Injury and poisoning	2008	4.87	570	24.52	8.93	960	41.29	9.48	795	34.19
	2009	3.86	455	14.95	8.38	904	29.71	16.55	1684	55.34
	2010	27.66	3282	38.34	19.53	2121	24.78	30.62	3157	36.88
	2011	37.06	4422	34.29	35.77	3909	30.32	43.72	4564	35.39
	2012	48.34	5787	36.49	46.69	5111	32.22	48.67	4963	31.29

^a^The mortality rate of neonatal refers to per 1, 000 (sic.) persons, others were per 100, 000 (sic.) persons

^b^Due to the different coverage of death surveillance, the data for cerebrovascular, cardiovascular, and injury and poisoning can’t be compared across 2008–2009 & 2010–2012

**Table 2:** The live birth and Population among different socio-economic groups, Chongqing, 2008 to 2012

Year	Less developed area				Relatively developed area				Developed area			
	Live births		Population		Live births		Population		Live births		Population	
	n	%	n	%	n	%	n	%	n	%	n	%
2008	116607	39.47	11706212	35.94	100334	33.96	10754806	33.02	78525	26.58	10109403	31.04
2009	123335	41.30	11795958	36.02	95456	31.96	10787808	32.93	79865	26.74	10172290	31.05
2010	98195	33.14	11864897	35.92	119746	40.41	10860515	32.88	78355	26.44	10309087	31.20
2011	111281	37.58	11930821	35.83	96281	32.52	10927458	32.82	88531	29.90	10439861	31.35
2012	115373	36.89	11972112	36.15	101056	32.31	10946535	33.05	96348	30.80	10197614	30.80

**Table 3:** Concentration index for EMS need indicators, Chongqing, 2008 to 2012

Year	Maternal	Neonatal	Cerebrovascular	Cardiovascular	Injury and poisoning
2008	−0.121	−0.092	0.446	0.319	0.252
2009	−0.136	−0.046	0.455	0.329	0.301
2010	−0.128	−0.161	0.106	0.185	0.020
2011	−0.096	−0.102	0.166	0.156	0.036
2012	−0.213	−0.094	0.108	0.101	0.001

Recent changes (2008 to 2012) for the maternal deaths declined steadily among all economic groups, and the neonatal deaths had a generally downward trend, particularly in the less developed area. For mortality of Maternal and Neonatal, the less developed area was 2.98 and 1.53 times of developed area (Table 1). In contrast, we documented heightened mortality rates in cerebrovascular, cardiovascular, injury and poisoning-related cases. The increase was particularly in developed area, and the mortality for cerebrovascular, cardiovascular in developed area were 1.625 and 1.59 times of less developed area. There are huge differences in the mortality rate of cerebrovascular, cardiovascular, injury and poisoning between 2008–9 and 2010–12—all increased sharply in the years from 2009 to 2010. The cause of these discrepancies was mainly that the death surveillance did not cover all districts until 2010, previously carrying out the work in only a small part of the country in Chongqing city.

Table 3 shows that, for maternal and neonatal deaths, the CI values were negative and ranged from −0.213 to −0.096, −0.161 to −0.046 respectively, indicating these undesirable health outcomes were concentrated in less developed area. The absolute value of CI for maternal deaths was 1.39 times of neonatal, and it was increasing over time, which meant the gap of equity was gradually strengthened among groups. For neonatal deaths, the magnitude of the CI was modest, and there was almost no change in 5 years. For cerebrovascular deaths, the absolute value of CI was 1.22 and 2.18 times of cardiovascular, injury and poisoning deaths, and the absolute value of CI for cardiovascular deaths was 1.78 times of injury and poisoning deaths. The CI all declined over time as a whole, indicating the gap of equity was significantly decreasing.

### Equity in EMS utilisation

Table 4 summaries the disparity in EMS utilisation, as reflected by the CI. According to the rate of utilisation, it was declined over time in less developed area, while in other areas it changed slightly. However, we found the sharp decreasing of emergency visits from 2011 to 2012 for less developed area and relatively developed area was very unusual. In Chongqing City, there was merger of two districts, and the main decreasing is there were two districts we could not find the emergency room visits in Chongqing Health Statistical Year Book both in less developed area and relatively developed area.

**Table 4:** Emergency utilisation and concentration index among different socio-economic groups, Chongqing, 2008 to 2012

Year	Less developed area			Relatively developed area			Developed area			CI
	Rate of utilisation	Emergency room visits		Rate of utilisation	Emergency room visits		Rate of utilisation	Emergency room visits		
	%	n	%	%	n	%	%	n	%	
2008	5.22	610990	20.30	6.33	580551	19.30	19.18	1817255	60.40	0.296
2009	4.56	538024	16.41	7.44	672410	20.51	21.42	2067679	63.08	0.340
2010	4.83	573213	16.40	7.53	730773	20.92	22.99	2189747	62.68	0.336
2011	4.25	506580	14.60	8.65	785406	22.64	21.46	2176989	62.76	0.347
2012	2.80	335775	11.18	6.38	564463	18.79	21.99	2103263	70.03	0.423

*CI* concentration index

The CI values ranged from 0.296 to 0.423 and increased over time, indicating patients in developed area were more likely to use emergency, the overall equality was lower, and the trend of equity showed an enlarged gap.

### Equity in distribution of related EMS resources

Table 5 shows the distribution of facilities, physicians and nurses. The overall results were as follows: per 100,000 people with emergency institutions were 0.209, 0.274 and 0.716; per 1000 people with emergency physicians were 0.018, 0.032 and 0.082, per 1000 people with nurses were 0.035, 0.070 and 0.096. The numbers were consistently higher in the developed areas.

**Table 5:** The distribution of facilities, physicians and nurses among different sample groups, Chongqing, 2013

Group	Facilities^a^			Physicians^b^			Nurses		
	n	%	Distribution (Per 100, 000 persons)	n	%	Distribution (Per 1, 000 persons)	n	%	Distribution (Per 1, 000 persons)
Less developed	25	19.53	0.209	30	10.87	0.018	59	14.46	0.035
Relatively developed	30	23.44	0.274	44	15.94	0.032	111	27.21	0.070
Developed	73	57.03	0.716	202	73.19	0.082	238	58.33	0.096

^a^Facilities is a total of emergency centers, stations and the departments with emergency ability in and out of networks among groups

^b^Physicians and nurses are full-time and part-time, pre-hospital and in-hospital in each sample area

Table 6 apparently shows the equity in resources distribution. For the G in population distribution of facilities, physicians and nurses were 0283, 0310 and 0.187, which showed a relative equity. The geographic distribution of related resources was 0.472, 0.516 and 0.412 respectively, indicating a big gap between groups.

**Table 6:** Gini coefficient in population and geographic distribution of facilities, physicians and nurses, Chongqing, 2013

Indicator	Resources	Less developed area		Relatively developed area		Developed area		G
		n	%	n	%	n	%	
Population^a^	Facilities	1197 2112	36.15	1094 6535	33.06	1019 7614	30.79	0.283
	Physicians	170 8908	30.75	138 2914	24.88	246 6501	44.37	0.310
	Nurses							0.187
Geographical area	Facilities	4 4981	54.61	2 3294	28.28	1 4089	17.11	0.472
	Physicians	7045	48.01	3739	25.48	3889	26.51	0.516
	Nurses							0.412

*G* Gini coefficient

^a^The population and geographic area of facilities are a total of each group, but physicians and nurses are a total of two sample areas in each group

## Discussion

By using the data for a 5-year period, we documented not only a stable decline in the maternal and neonatal deaths in Chongqing city, but also an enlarging gap of equity between poor and rich areas for maternal deaths. The results were similar to other studies [32, 33]. For example, the research on equity of maternal mortality in China from Wang B, et al. showed that from 2000 to 5, the maternal deaths were mainly concentrated in the western provinces (less developed areas), and during a 6-year period, inter-regional and inter-provincial equity has not significantly improved. The relatively inequitable distribution of maternal and neonatal deaths in Chongqing can be explained by the regional differences in the development of economic and the government investment in health infrastructure. In poor areas, the EMS capacity is not strong: the physicians and nurses are less experienced, lack training in emergency skills [34]. Congested traffic extends the emergency response time, etc. Meanwhile in rich areas, there are often several top three hospitals within 5 km. The distance to the nearest health institutions is less than 1 km, taking patients less than 20 min [34]. The professional undertakes significant scientific projects and teaching missions, etc. Consequently, it is obvious that people in these areas are better accessibility for EMS.

The gap of cerebrovascular, cardiovascular, injury and poisoning-related deaths among groups was reduced, based mainly upon the rapid development of social economy and transportation. On the one hand, the majority of residents in rural areas is not dominated by agriculture, but for workers from rural to urban, the improvement of living conditions and the change of living habits may increase the risk of cardiovascular diseases. And given the lower education, the awareness of preventing cardiovascular diseases is weaker than for urban residents. On the other hand, to support the construction of new rural areas and break the traffic bottleneck, the national policy of implementing access roads for every village had solved most of farmers’ travel difficulties. Simultaneously, more and more villagers take motorcycle as their primary mode of transport. However, they have poor driving skills, and they tend to be unfamiliar with and to disobey the traffic rules, and these factors may be the major causes of motor vehicle accidents increasing in rural areas.

The use of EMS was lower and fairly inequitable across groups. The 2001 National Health Interview Survey in Taiwan showed that the CI for utilisation of EMS was 0.1188 [35], much lower than in Chongqing city. In Taiwan, low income groups bear relatively low premium burden, and in order to avoid delayed medical treatment, it provided equal opportunities to receive quality healthcare. While in Chongqing city, ambulance is regarded as the best way for desperate patients to reach the nearest medical institutions, but it is expensive and cannot be reimbursed by health insurance. This may reduce the willingness to use it, especially for rural residents of lower income. Moreover, given the lack of emergency doctors and drivers in some township hospitals, the ambulance simply can not provide services.

This study has found a negative correlation in EMS needs and provision. In spite of a negative CI trend for EMS needs, surprisingly, the utilisation of emergency was not, as a trend, fairly centralised among poor areas, but actually showed a pro-rich character, and the gap of inequity even continued expanding. Notably, the patients in poor areas with higher needs of EMS were less likely to access emergency treatment.

These results illustrated that EMS needs, utilisation and resources distribution among areas is not equitable, and it was better provided in rich areas than in poor areas. Thus, we should take measures to make EMS more equitable and resolve the worst health problems. According to the findings of this study, we could start with the following aspects:

First, promote the capability in emergency treatment and improve human resources in poor areas. On the one hand, the physicians should strengthen the on-site first aid techniques, especially for intubation, traumatic brain injury, defibrillation, fracture and hemostasis. The nurses should grasp cardiopulmonary resuscitation, and have a good command of using emergency equipments and drugs, such as cardiopulmonary resuscitation machine, ventilator, electrocardiograph and uterotonics, etc. Human resources are the material basis of treatment capacity. The government ought to introduce adequate and experienced professionals in rural health care by increasing the establishments, giving extra subsidies and other preferential policies.

Second, perfect the allocation of emergency institutions. EMSS requires precise information and supply systems to monitor and rescue the patient that needs EMS. The shortage and unequal distribution of Emergency institutions (for the allocation of facilities, the developed area were 3.43 and 2.61 times of less developed area and relatively developed area) increase the transport time for patients and diminish their chances of survival. In poor areas, it is imperative that EMSS should be emphasized in primary health institutions, building communication with qualified to improve the accessibility for EMS. Moreover, we need to adjust the distribution of emergency centres, stations and hospital networks to change the institutions in idle states and address the imbalance.

Last, develop the economy to narrow the utilisation gap in poor areas. According to several studies, economy is the principal factor affecting the equity of heath service, both in urban and rural areas [4, 17, 19]. It shows the supplying capacity of one person, district and country, and the economic gain could create more opportunity to stimulate equity. As people in poor areas show lower use of EMS, we should promote the economy to give low-income patients better access to them. Then the institutions could provide adequate human resources to reduce the utilization gap among groups.

### Study limitations

Several limitations of this study must be mentioned. First, although the related mortality could be considered as indicators of EMS needs, a few cases were undiscovered and remained off the records due to social and individual factors, especially the death and burial in some remote rural areas. In addition, the quality of data reported by institutions differed by districts, which may have underestimated the equity. However, this study took a broader perspective and used a cross-sectional approach, focusing on single disease to explore the relationship between needs and utilisation; we believe the data did reflect the trend of equity. Furthermore, five needs-related emergency room visits could not be extracted from the whole data that could better reflect the utilisation of EMS.

## Conclusions

EMS is inequitable among regions in Chongqing city. Major challenges are in connection with equity between EMS needs and provision, particularly in poor areas. The emergency capability and economy are two major determinants of it. Key policies and actions should be at the top of the government agenda to reduce the gaps.
